# The evolution of pattern camouflage strategies in waterfowl and game birds

**DOI:** 10.1002/ece3.1482

**Published:** 2015-04-22

**Authors:** Kate L A Marshall, Thanh-Lan Gluckman

**Affiliations:** 1 Department of Zoology, University of CambridgeCambridge, CB2 3EJ, UK; 2 Department of Zoology, University of MelbourneParkville, Victoria, 3010, Australia; 3 Department of Animal and Plant Sciences, University of SheffieldWestern Bank, S10 2TN, UK

**Keywords:** Background matching, bimodal signal, birds, camouflage, communication, evolution

## Abstract

Visual patterns are common in animals. A broad survey of the literature has revealed that different patterns have distinct functions. Irregular patterns (e.g., stipples) typically function in static camouflage, whereas regular patterns (e.g., stripes) have a dual function in both motion camouflage and communication. Moreover, irregular and regular patterns located on different body regions (“bimodal” patterning) can provide an effective compromise between camouflage and communication and/or enhanced concealment via both static and motion camouflage. Here, we compared the frequency of these three pattern types and traced their evolutionary history using Bayesian comparative modeling in aquatic waterfowl (Anseriformes: 118 spp.), which typically escape predators by flight, and terrestrial game birds (Galliformes: 170 spp.), which mainly use a “sit and hide” strategy to avoid predation. Given these life histories, we predicted that selection would favor regular patterning in Anseriformes and irregular or bimodal patterning in Galliformes and that pattern function complexity should increase over the course of evolution. Regular patterns were predominant in Anseriformes whereas regular and bimodal patterns were most frequent in Galliformes, suggesting that patterns with multiple functions are broadly favored by selection over patterns with a single function in static camouflage. We found that the first patterns to evolve were either regular or bimodal in Anseriformes and either irregular or regular in Galliformes. In both orders, irregular patterns could evolve into regular patterns but not the reverse. Our hypothesis of increasing complexity in pattern camouflage function was supported in Galliformes but not in Anseriformes. These results reveal a trajectory of pattern evolution linked to increasing function complexity in Galliformes although not in Anseriformes, suggesting that both ecology and function complexity can have a profound influence on pattern evolution.

## Introduction

Visual patterns typically evolve to enhance their function in camouflage and/or communication (Endler 1978; Bradbury and Vehrencamp 1998; Kenward et al. 2004). A broad sample of the literature spanning over 80 studies demonstrates that, although visual patterns generally have a camouflage function, it is dependent on pattern type and context (Table1; Table S1). For example, primary camouflage patterns prevent detection while stationary (“static camouflage”) (e.g., Hanlon and Messenger 1988; Hemmi et al. 2006) whereas secondary defense patterns prevent capture during movement (“motion camouflage”) (e.g., Brodie 1992; How and Zanker 2013). In addition to a camouflage function, some pattern types function in intraspecific visual communication to attract mates and/or intimidate rivals (e.g., Petrie et al. 1991; Swaddle and Cuthill 1994; Roulin et al. 2010). Therefore, patterns can have single or dual functions in camouflage and visual communication, which may have consequences on the evolutionary history of different pattern types.

**Table 1:** The number of species for which empirical, comparative, and correlational evidence has demonstrated the function of irregular or regular patterns in camouflage and/or communication, spanning vertebrates, and invertebrates, as well as terrestrial and aquatic species (*see* Table S1 for source studies)

	Irregular	Regular
Camouflage	8	7
Communication	1	7

**Figure 1:**
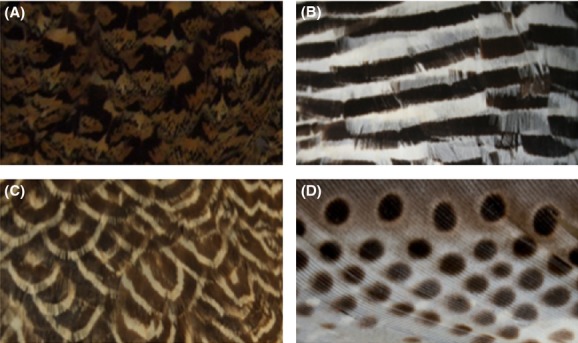
Irregular and regular plumage patterns found in birds. Irregular: (A) mottled plumage in a female sharp-tailed Grouse (*Tympanachus phasianellus*); regular: (B) barred plumage in a male Andean Grouse (*Chloephaga melanoptera*), (C) scaled plumage in a male Falcated duck (*Anas falcata*), (D) spotted plumage in a male Great Argus (*Argusianus argus*). Photographs were taken by Thanh-Lan Gluckman. Copyright of Museum Victoria.

Patterns that function only in static camouflage are likely to consist of heterogeneous pigmentation, such as mottled plumage and stipples (hereafter referred to as irregular patterns; Fig.1A). Irregular patterns are often effective in static camouflage strategies via background matching (e.g., Stoner et al. 2003; Merilaita and Lind 2005; Lovell et al. 2013) and disruptive camouflage (e.g., Caro 2005; Schaefer and Stobbe 2006; Stevens et al. 2009; Troscianko et al. 2013; Webster et al. 2013) (Table1; Table S1). Moreover, regular patterns that repeat the same motif, such as scales, bars, and spots (Fig.1B–D), appear to be detrimental to the effectiveness of static camouflage (e.g., Merilaita and Lind 2005; Stevens et al. 2011; Dimitrova and Merilaita 2012), which further suggests that irregular patterning is important in the camouflage of stationary animals.

While irregular patterns are linked to static camouflage, highly contrasting patterns that regularly repeat the same motif (hereafter referred to as regular patterns e.g., bars, stripes; Fig.1) typically function in motion camouflage. Regular patterns act as a secondary defense to prevent capture by predators of moving (escaping) prey (Table1; Table S1; e.g., Jackson et al. 1976; Brodie 1989; Zanker and Walker 2004; Stevens et al. 2008, 2011; How and Zanker 2013; Hughes et al. 2014), with some exceptions to this general trend (e.g., Godfrey et al. 1987; Allen et al. 2011; Kjernsmo and Merilaita 2012; Santer 2013). Much evidence has indicated that regular patterns also function in intraspecific communication, largely because the repetition of information in a regular pattern can increase the likelihood that a signal will be received (Table1; Table S1; e.g., Petrie et al. 1991; Swaddle and Cuthill 1994; Omland 1996; Roulin 1999; Kenward et al. 2004; Gluckman and Cardoso 2010; Roulin et al. 2010; Muck and Goymann 2011). Taken together, previous work suggests that irregular patterns facilitate static camouflage whereas regular patterns have a dual function in both motion camouflage and communication.

Additionally, some species possess both irregular and regular patterns over different parts of the body and/or between the sexes (“bimodal” patterning). While the distribution of patterning between the sexes is not always indicative of function, as recently shown in birds (e.g., Burns 1998; Clutton Brock 2009; Roulin et al. 2010, 2013; Nordeide et al. 2013; Gluckman 2014), spatial separation of different types of patterns over the body may combine static and motion camouflage as an enhanced form of concealment or a signal partitioning strategy for simultaneous camouflage and communication (Endler 1978, 1980, 1987; Stuart-Fox and Ord 2004; Oliver et al. 2009; Gluckman and Cardoso 2010; Zylinski et al. 2011; Chen et al. 2012; Garcia et al. 2013). Therefore, depending on their ecology and life history, species that exhibit bimodal patterns are likely to gain adaptive benefits compared to species exhibiting irregular or regular patterns with only single or dual functions.

**Figure 2:**
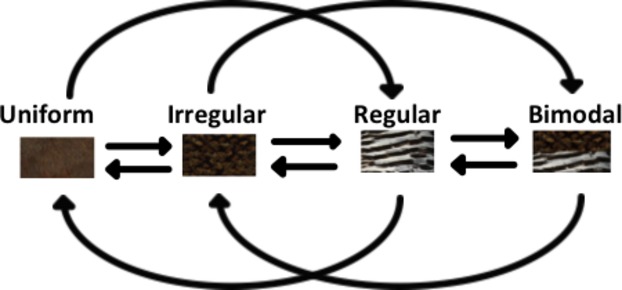
Hypothesis of plumage pattern evolution. Both irregular and regular patterns evolve first followed by bimodal pattern phenotypes consisting of both irregular and regular patterning. Conversely, bimodal patterning must transition via the singular regular or irregular types before being entirely lost.

Little is known about the relative importance of these different pattern types (and their related functions) and the order in which they have evolved, and how their prevalence and evolutionary history have been influenced by ecology. Therefore, we addressed these questions in two bird groups – waterfowl (Anseriformes) and game birds (Galliformes) – that have distinct life histories and exhibit the described pattern types (e.g., irregular mottled plumage Fig.1A; regular barred plumage Fig.1B; regular spotted plumage Fig.1C). Anseriformes typically inhabit aquatic open habitats and escape by flight, whereas Galliformes inhabit terrestrial closed environments (e.g., woodland) and often use a “sit and hide” strategy when threatened (del Hoyo et al. 1992, 1994). In each group, we measured the relative frequency of each of the three pattern types and used Bayesian comparative modeling to trace their evolutionary trajectory. Our first hypothesis addressed the relative frequency of each pattern type in each group. Given their different life histories, we hypothesized that Anseriformes would predominantly possess regular (motion-based) camouflage patterns while Galliformes would more frequently have irregular (static-based) camouflage patterns or bimodal patterning. That is, we expected regular patterns to be more common in waterbirds and irregular patterns to be more common in game birds due to selection favoring these patterns in relation to their adaptive function. Our second hypothesis addressed the evolutionary trajectory of the different pattern types. Specifically, we hypothesized that, with each evolutionary transition, patterns would evolve to be more complex in function. That is, over the course of evolution beginning with an ancestral state of uniform coloration, patterns should develop from having a single function (irregular) to having dual functions (regular) to having multiple functions (bimodal). Specifically, we predicted that bimodal/signal partitioned phenotypes would be the most derived state (Fig.2) and must first evolve via singular irregular or regular patterns before being lost (Bro-Jørgensen 2010). Accordingly, we predicted that direct evolutionary transitions from uniform coloration to bimodal patterning, as well as backward evolutionary transitions from bimodal patterning to uniform coloration, would not occur.

## Materials and Methods

### Data collection

We used published phylogenies with branch length information: Anseriformes – Gonzalez et al. (2009) which covers 118 spp; Galliformes – Kimball et al. (2011) which covers 170 spp. Together, they include 63% of species belonging to both orders combined and all families are represented. To classify patterning for each species (nominate subspecies were selected where applicable), we referred to field guides as they describe the majority of the visual traits contributing to avian phenotypes (field guide references are provided in the Supporting Information).

In most of the study species, information about their sociality, predators, and specific antipredator behaviors (e.g., the distance required to cause a reaction, predator approach angle) is lacking. In addition, the prevailing view that sexual selection governs sexual dimorphism has been challenged in multiple studies and an understanding of female plumage is in its infancy (Irwin 1994; Burns 1998; Amundsen 2000; Hofman et al. 2006; Kraaijeveld et al. 2007; Clutton Brock 2009; Cardoso and Mota 2010; Roulin et al. 2010, 2013; Roulin and Ducrest 2011; Nordeide et al. 2013; Gluckman 2014). Given that there is a large body of evidence linking pattern type with function, we focus solely on the type of pattern present in each species regardless of the sex.

We assigned the character state of both sexes of each study species with uniform coloration, irregular, regular, and bimodal patterns (Figs.1, 2; Table2; Table S2, S3). Plumage pigmentation that is heterogeneous without a well-defined motif was scored as irregular patterning (Fig.1A). Patterns consisting of a regularly repeating motif – bars (Fig.1B), scales (Fig.1C), or spots (Fig.1D) – were scored as regular patterns. From our plumage data, if a phenotype consisted of both regular and irregular patterns it was classified as bimodal. Species in which only one sex had patterns and the other had uniform coloration were classified as having patterns, as the focus of this study is signal evolution rather than sexual dimorphism (e.g., Gluckman 2014). Where species exhibited variable patterns between molts we used the breeding plumage.

**Table 2:** The proportion of species with each type of pattern in Anseriformes and Galliformes and their subfamilies as well as tribe or subclade, where applicable. The percentage of species with each type of pattern is calculated at the Order, subfamily and Tribe or subclade as per the phylogenetic relationships published in Gonzalez et al. (2009) for Anseriformes and Kimball et al. (2011) for Galliformes

Order	Subfamily	Tribe or subclade	Uniform %	Irregular %	Regular %	Bimodal %
Anseriformes			33	8	43	16
	Anatinae		28	12	45	15
		Anatini	11	17	52	20
		Aythyini	31	13	50	6
		Tadornini	55	–	27	18
		Mergini	67	–	25	8
	Anserinae		52	–	34	14
		Anserini	43	–	36	21
		Cygnini^1^	75	–	25	–
		Oxyurini^1^	43	–	43	14
	Dendrocyginae		–	–	50	50
Galliformes			21	11	33	35
	Megapodidae		80	13	7	–
	Cracidae		61	13	26	–
	Numididae		20	–	80	–
	Odontophoridae		13	–	25	63
	Arborophilinae^1^		13	–	50	38
	“Core” Phasianidae^1^		6	13	35	46
		Turkeys, grouse, “true” pheasants and allies	9	5	32	54
		Junglefowl, bamboo partridges, and quail-francolins	0	31	13	56
		Old world quail, partridges, partridge-francolins, and snowcocks	4	25	38	33
		Peacock-pheasants	11	–	67	22
		Peafowl	–	–	67	33
		Argus pheasants	–	–	100	–

1 Following Gonzalez et al. (2009) Cygnini includes *Malacorhynchus membranaceus*. Following Kimball et al. (2011) Oxyurini includes *Biziura lobata* and the Phasianidae are split into the Arborophilinae and “core” Phasianidae.

We tabulated the number of species with each type of pattern. To account for phylogeny, we present this data as the proportion of species with each pattern type per order, subfamily as well as “tribe” or subclade, as these proportions are independent between families (Gluckman and Cardoso 2010). In the majority of species classified as bimodal, both sexes have the same phenotype (51%). Of the remaining 49% of bimodal species, 37% had singular irregular or regular patterns in one sex and bimodal patterns in the other sex, and 12% had regular patterns in one sex and the other sex had irregular patterns.

### Estimating plumage pattern evolution

We hypothesized that with each evolutionary transition, patterns would evolve to be more complex in function. That is, patterns should evolve in a predictable direction from having a single function (irregular) to having dual functions (regular) and that bimodal phenotypes, being the most derived state, must first evolve via singular irregular or regular patterns (Fig.2). Conversely, a lack of support for this hypothesis would show direct evolutionary transitions from uniform coloration to bimodal patterns and an absence of transitions from uniform coloration to singular irregular and regular patterns. In addition, if there is no directionality in plumage pattern evolution toward increasing complexity, or no order in plumage pattern evolution irrespective of our hypothesis, the full (null) model, where every evolutionary transition between all pattern states occurs, would be supported above all other models of evolution.

To test this hypothesis, we traced the evolutionary history of plumage patterns by estimating the rate at which plumage evolves between uniform coloration, irregular, regular, and bimodal patterns in each group separately using the Reversible Jump Markov chain Monte Carlo Multistate option in BayesTraits v.1 (Gelman et al. 2003; Pagel et al. 2004; Pagel and Meade 2006). This approach avoids a dependency on ancestral state reconstruction, as the estimates are produced from a most recent common ancestor approach.

Under Markov chain Monte Carlo (MCMC), plumage patterns can repeatedly evolve between any pattern state. The Markov chain estimates the rate of change between pattern states, conditioned on the values at the tips, in proportion to their probability. In successive steps, the Markov chain proposes new rate parameter values, resulting in a posterior sample distribution of coefficients of rates of change. Thus, the sample distribution is composed of models of plumage pattern evolution comprised of rate coefficients (Pagel et al. 2004). To avoid over parameterization, we employed Reversible Jump MCMC (RJMCMC).

RJMCMC searches the posterior distribution of model parameters to integrate rate restrictions. Therefore, we did not constrain any rate parameters to equal 0, allowing incremental and nonsequential changes to occur and ensuring that the analysis is conditional on the data rather than a priori predictions (Gluckman 2014). Potential models of pattern evolution are distinct from the most probable model of pattern evolution, as the latter is derived by statistically evaluating the posterior sample distribution. The composition of each model of pattern evolution in the sample distribution is comprised of a unique combination of transition rate parameters that are sampled as free parameters with positive values or with values fixed to zero. We interpreted rate parameters that are fixed to zero as an evolutionary transition that does not occur, and rate parameters with a positive value were considered evidence of an evolutionary transition that does occur. Therefore, each unique model of pattern evolution qualitatively consists of transitions that occur, as well as transitions that do not. If there were no directionality in plumage pattern evolution, and transitions between patterns occurred at random in both forward and backward directions, the full (null) model would be supported.

### Statistical analysis

We report on the average probability of each type of pattern being the ancestral state. We then statistically analyzed the sample distribution of models to examine support for the null model, as well as conducting model comparison. By statistically evaluating the entire sample distribution of models of pattern evolution, we compared each unique model with all other models. To qualify the probability of each evolutionary transition between pattern states not occurring, or occurring, while accounting for model variation in the sample distribution, we used multimodel inference (Burnham and Anderson 2002; Gluckman 2014).

We evaluated which models of pattern evolution are visited more than expected by chance using Bayes factors (BF) derived from the prior and posterior odds. The prior probability of encountering each unique model of pattern evolution was calculated using binomial numbers for transitions that are fixed to zero, and bell numbers for transition rates that have a positive value (Table S4; *see* Currie et al. (2010) for detailed explanation). The posterior probability of each model of pattern evolution was calculated as model frequency/the sample distribution. The BF for each unique model of pattern evolution was posterior probability/prior probability. A BF of ≥2 is positive evidence and was used as a threshold to derive a top model set (Kass and Raftery 1995; Burnham and Anderson 2002).

**Figure 3:**
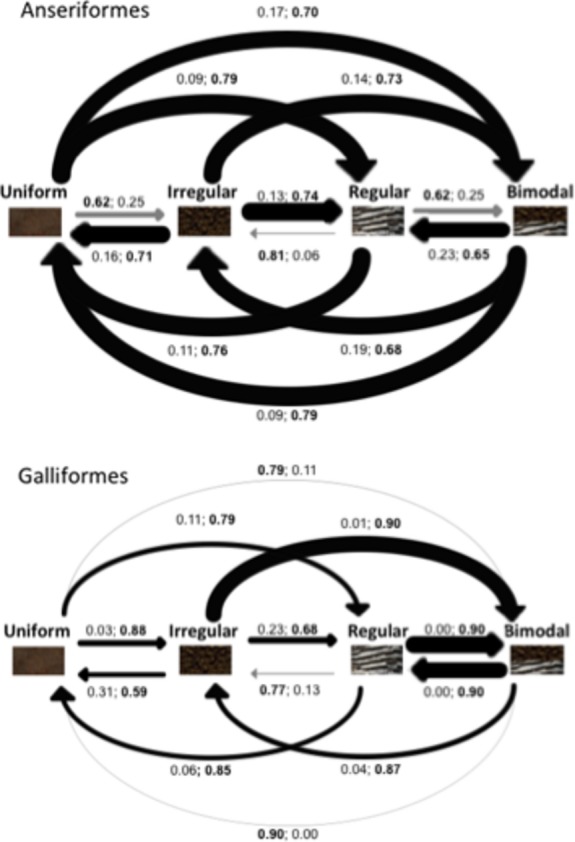
The most probable model of plumage pattern evolution in Anseriformes and Galliformes derived from the top model set. The width of each transition is proportional to its average rate of transition. Each evolutionary transition is depicted with its marginal probability of not occurring and occurring, respectively. A gray line indicates a transition that probably does not occur, and a black line indicates a transition that probably occurs. The total of the marginal probability of occurrence and nonoccurrence does not add up to 1, as these are the transitions of the top model set rather than the entire posterior sample distribution.

As a consequence of model uncertainty, some rate parameters vary widely in their values. To incorporate this variation, we calculated the marginal probability (MP) from the entire sample distribution, for example, MP - (unique model/sample distribution size). To summarize our findings, we cumulatively added the MP of each transition being fixed to 0 or a positive value, in the top model set, for comparison (Burnham and Anderson 2002). For example, the MP of a transition from uniform coloration to regular patterns *not* occurring in Anseriformes is 0.09, and the MP of occurring is 0.79, and therefore probably occurs (Fig.3). The sum of the MP of a transition being fixed to zero and being sampled as a free parameter with a positive value rarely equals 1, as this only occurs in the absence of variation in the sample distribution.

### Model settings

To reduce uncertainty in choice of model priors, we used the hyperprior option and seeded the interval for the prior seed distribution using a Bayes estimator approach with a gamma distribution (Pagel et al. 2004). The first 50,000 iterations of each Markov chain were discarded as burn-in. To ensure stable results, we ran the analysis four times for 10,050,000 iterations per chain, for each group. Convergence of chains was indicated by a harmonic mean that varied <1 lnHM over each analysis. We modified the ratedev parameter to maintain an acceptance rate between 0.20 and 0.40. We sampled every 1000th model, resulting in a posterior sample of 40,000 models per order (*n* - 10,000 × 4). Autocorrelation was present, and we further sampled every 20th model resulting in 2000 models per order (Anseriformes Ljung-box *P* - 0.129, Galliformes Ljung-box *P* - 0.319). Further details are available in the Supporting Information.

## Results

All types of patterning are present in both orders (Table2; Figs. S2, S3). Species that have uniform coloration are the minority in Anseriformes (33%) and Galliformes (21%) (Table2). In Anseriformes, uniform coloration and regular patterns are present in all tribes. However, irregular patterns have only evolved in the more derived species of Anatinae (*Anatini* and *Aythyini*), whereas bimodal patterns have evolved in all tribes except Cygnini (Fig. S2). In the basal Anseriformes group, Dendrocygninae, the only pattern phenotypes are regular and bimodal patterns. In Galliformes, the more basal lineages frequently possess uniform coloration (e.g., Megapodidae and Cracidae; Fig. S3). Irregular patterns are found in the basal Megapodidae, and only re-evolved in the derived Arborophilinae and are absent in the other galliform families. Regular and bimodal patterns have predominantly evolved in the more derived core Phasianidae. Of the six subclades of the core Phasianidae, three subclades only have regular or bimodal patterns – peacock-pheasants, peafowl, and the argus pheasants (Table2; Fig. S3).

The most probable ancestral plumage in Anseriformes was equivocal (*P* - 0.25 for each pattern in every model proposed), whereas for Galliformes it was uniform coloration (uniform MP - 0.80, all other patterns MP ≤0.10). The top model set was composed of 82 unique models in Anseriformes and 54 unique models in Galliformes. There was no support for the full model in the entire posterior sample distribution of models (Anseriformes null frequency - 0; Galliformes null frequency - 3, BF - 0.01).

There was evidence for directionality in the models of pattern evolution in that some transitions occur and some do not (Fig.3). In Anseriformes, regular and bimodal patterns evolved from uniform coloration with a high average rate of transition and irregular patterns evolved subsequent to the evolution of regular and bimodal patterns. In Galliformes, irregular or regular patterns evolved from uniform coloration and bimodal patterns evolved subsequent to the evolution of irregular and regular patterns. In both orders, bimodal patterns evolve from irregular patterns with a high average rate of transition whereas a transition to bimodal patterns from regular patterns only occurs in Galliformes. In addition, irregular patterns evolve into regular patterns, but not the reverse, whereas bimodal patterns evolve into either regular or irregular patterns. In Anseriformes, a transition between uniform coloration and bimodal patterning can occur via multiple pathways with a high rate of transition, whereas in Galliformes, a transition between uniform coloration and bimodal patterns can only evolve via singular irregular as well as regular patterns.

## Discussion

We investigated the relative frequency and evolutionary trajectory of three different plumage pattern types (irregular, regular, and bimodal) in waterfowl (Anseriformes) and in game birds (Galliformes), and the potential influence of their distinct life histories and ecology on pattern evolution. Based on a broad survey of the literature across aquatic and terrestrial invertebrates and vertebrates, we revealed that each type of pattern is typically associated with different functions that vary in number and complexity (Table1, Table S1). Specifically, in at least eight species (including amphibians, insects, crabs, cephalopods, and birds [eggs]) and in prey–predator simulations with human “predators”, irregular patterns have been shown to function in only static camouflage (e.g., Hanlon and Messenger 1988; Stoner et al. 2003; Caro 2005; Merilaita and Lind 2005; Hemmi et al. 2006; Schaefer and Stobbe 2006; Stobbe and Schaefer 2008; Stevens et al. 2009; Lovell et al. 2013; Troscianko et al. 2013). Moreover, empirical and correlational evidence in at least seven species (comprising fish, snakes, mammals, and cephalopods) and prey–human predator simulations have demonstrated that regular patterns function in motion camouflage (Jackson et al. 1976; Lindell and Forsman 1996; Zanker and Walker 2004; Stevens et al. 2008, 2011; Zylinski et al. 2009; Scott-Samuel et al. 2011; Hall et al. 2013; von Helversen et al. 2013; How and Zanker 2013; Hughes et al. 2014), while regular patterns can additionally facilitate intraspecific communication, as shown in at least seven species including birds and fish (e.g., Swaddle and Cuthill 1994; Omland 1996; Roulin 1999; Siebeck 2004; Bortolotti et al. 2006; Roulin et al. 2010; Muck and Goymann 2011). Finally, bimodal patterns potentially provide all three functions, as shown in birds, *Bicyclus* butterflies and lizards (e.g., Stuart-Fox and Ord 2004; Oliver et al. 2009; Gluckman and Cardoso 2010; Chen et al. 2012; Garcia et al. 2013).

We found that all types of patterning were frequently represented in Anseriformes and Galliformes, but their distribution varied over each phylogeny. As predicted, in Anseriformes, regular patterns were most frequent, suggesting that they are the most important pattern type for this bird group. However, in Galliformes, bimodal and regular patterns were most frequent. Thus, despite the differences in ecology and life histories between the two groups, both had a relatively low frequency of irregular patterns and a bias toward patterns with dual or multiple functions. This suggests that selection tends to favor patterns that provide dual or multiple functions over static camouflage alone, irrespective of ecology or life history (Table2; e.g., Marshall 2000; Gluckman and Cardoso 2010; Muck and Goymann 2011) and that regular and bimodal pattern types are relatively beneficial to survival and reproduction. As animals generally require visual patterns that function in both camouflage and intraspecific communication, patterns with multiple functions would be more efficient and thus tend to be broadly favored by selection over patterns with a single function.

The high prevalence of regular patterns in Galliformes was an unexpected finding, given that they tend to use a stationary “sit and hide” antipredator strategy (del Hoyo et al. 1992, 1994), and would therefore theoretically benefit more from irregular patterns for static camouflage than from regular patterns for motion camouflage. However, as an exception to our assumption here (based on the general trend in the literature), it is possible that regular patterns in Galliformes also function in static camouflage, particularly if they are found in their visual background, as shown in some taxa (e.g., Godfrey et al. 1987; Allen et al. 2011; Kjernsmo and Merilaita 2012; Santer 2013). Moreover, game birds may escape by flight as a last resort so that motion camouflage provided by regular patterns might act as a secondary defense against predators (e.g., Brodie 1989; del Hoyo et al. 1992, 1994; Zanker and Walker 2004; Stevens et al. 2008, 2011; How and Zanker 2013; Hughes et al. 2014). These results imply that, while certain pattern types in animals may be typically associated with specific functions and ecological factors, fluctuating social and physical environments will inevitably produce exceptions to the general link between pattern type and function we report here.

Despite this, we note that static camouflage via irregular patterns may have been important early in the evolution of game birds, given that it most probably evolved first from the ancestral uniform state in comparison with regular patterning (Fig.3). While irregular and regular patterns were more likely to evolve first in Galliformes, bimodal patterns were more derived, which supports our hypothesized trajectory of increasing complexity in pattern function. This suggests that, as predicted, over the course of evolution patterns have evolved to increase function in Galliformes, starting from fewer functions (irregular/regular) and transitioning to the most complex function (bimodal), indicating strong selection for multiple functions via bimodal patterns (Table2). Bimodal patterns may have been favored by selection over regular patterns in game birds because of the potential costs that regular patterns incur to static camouflage (Merilaita and Lind 2005; Stevens et al. 2011; Dimitrova and Merilaita 2012) and their ability to provide both static and motion camouflage, as well as effective visual signals in their typically cluttered terrestrial habitats (Kenward et al. 2004). These results provide further evidence that patterns providing multiple functions are broadly favored by selection over those providing single functions. Moreover, our results indicate that pattern functions can increase in number and complexity over the course of evolution.

Conversely, in Anseriformes, regular or bimodal patterns evolved first from the ancestral uniform state, and a transition to bimodal patterning from regular patterning did not occur, which did not support our prediction of increasing pattern function with each evolutionary transition (Fig.2). Instead, these findings suggest that, in contrast to Galliformes, the evolutionary trajectory of waterfowl patterns is not linked with increasing function and that patterns offering dual or multiple functions (particularly regular patterns) have been an important strategy from early in their evolutionary history. Given that waterbirds typically escape predators by flight and occupy open aquatic habitats (del Hoyo et al. 1992, 1994), they are likely to have gained little adaptive benefit from irregular patterns offering a single static camouflage function. Instead, selection seems to have favored regular patterns that fulfill both the demands of motion camouflage and intraspecific signaling (e.g., Marshall 2000; Oliver et al. 2009; Gluckman and Cardoso 2010). Indeed, why have only one function when you can have two in one? However, we note that the dual role of regular patterns may depend on the signal location on the body as well as the viewing angle and visual capabilities of the receivers (Endler 1992), which warrants future investigation.

Overall, our findings suggest that consecutive evolutionary transitions in patterns can increase function, although not necessarily. Instead, over the course of evolution, selection should favor specific patterns that are adaptive (in relation to species’ life history and ecology) during each evolutionary transition. Since first evolving, the patterns of Galliformes birds have changed markedly in function, while Anseriformes patterns have shown little functional gain. This may be linked to the degree in which their respective social and physical environments have changed over evolutionary history, seeming to fluctuate much more in game birds than in waterfowl.

In summary, in two bird groups with differing life histories and ecology, we investigated the importance and evolution of different pattern types with typical functions varying in number and complexity. Our results suggest that patterns with dual/multiple functions (i.e., in camouflage and intraspecific communication) are generally favored by selection over patterns that have a less complex, single function in static camouflage. Nevertheless, the number and complexity of functions provided by different pattern types do not necessarily determine their evolutionary history. Species’ environments and life histories and the extent to which they fluctuate appear to have profound effects on pattern evolution that could override gradual increases in function. However, further work is needed to understand the link between pattern evolution and function. For example, little is known about how pattern evolution resolves the competing demands of camouflage and communication, whether the same pattern type has the same function in multiple species (with the exception of barred and spotted plumage patterns, e.g., Swaddle and Cuthill 1994; Roulin 1999; Gluckman and Cardoso 2010; Roulin et al. 2010), or what factors influence the extent to which selection favors particular types of patterns and their respective functions.

## Supporting Information

**Figure S1.** The evolution of singular and bimodal patterns in Anseriformes. Both singular and bimodal patterning is found in extant species. **Figure S2.** The evolution of singular and bimodal patterns in Galliformes. Both singular and bimodal patterning is found in extant species. **Table S1.** A representative sample of literature examining pattern type and pattern function in different contexts (stationary or moving), in birds, mammals, fish, reptiles, insects, cephalopods, crustaceans, and amphibians. **Table S2.** Character states across Anseriformes in taxonomic order. **Table S3.** Character states across Galliformes in taxonomic order. **Table S4.** Calculation of prior odds of seeing any particular model of evolution on the basis of the number of transition parameters (*see* Currie et al. 2010 for a detailed description of the calculations used).M
