# Supplementary material for: The evolution of pattern camouflage strategies in waterfowl and game birds
Source: Ecol Evol. 2015 Apr 22;5(10):1981–91. doi: 10.1002/ece3.1482 (PMC4449753; doi:10.1002/ece3.1482)
Supplement: Supplementary file 1 [file ece30005-1981-sd1.docx]

**
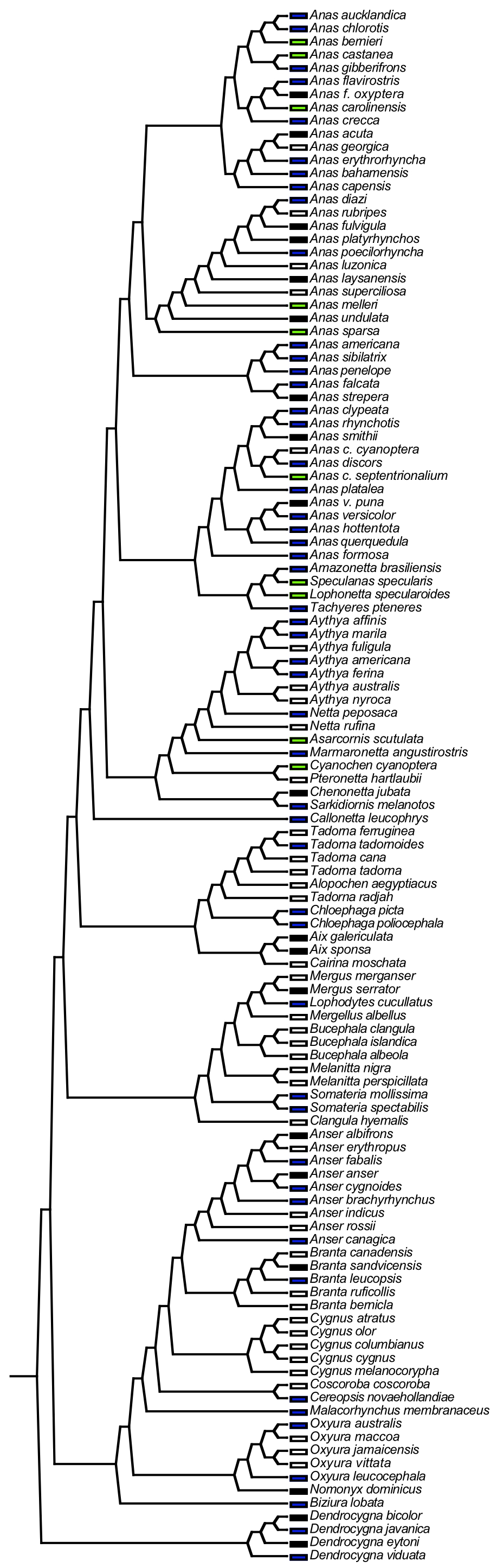
Online supplementary material**

The evolution of camouflage strategies in waterfowl and gamebirds

Marshall, K.L.A. and Gluckman, T-L.

**
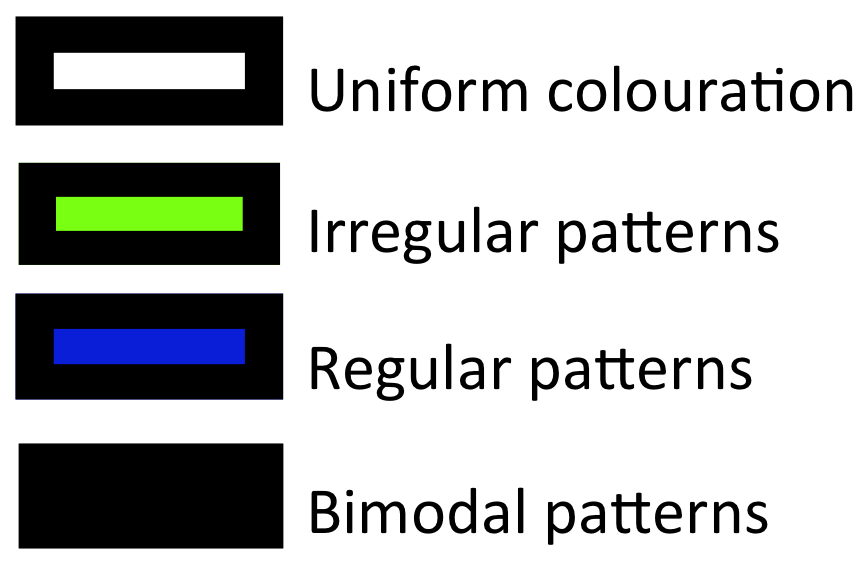
**

**Fig. S1.** The evolution of singular and

bimodal patterns in Anseriformes. Both

singular and bimodal patterning is found

in extant species.

**
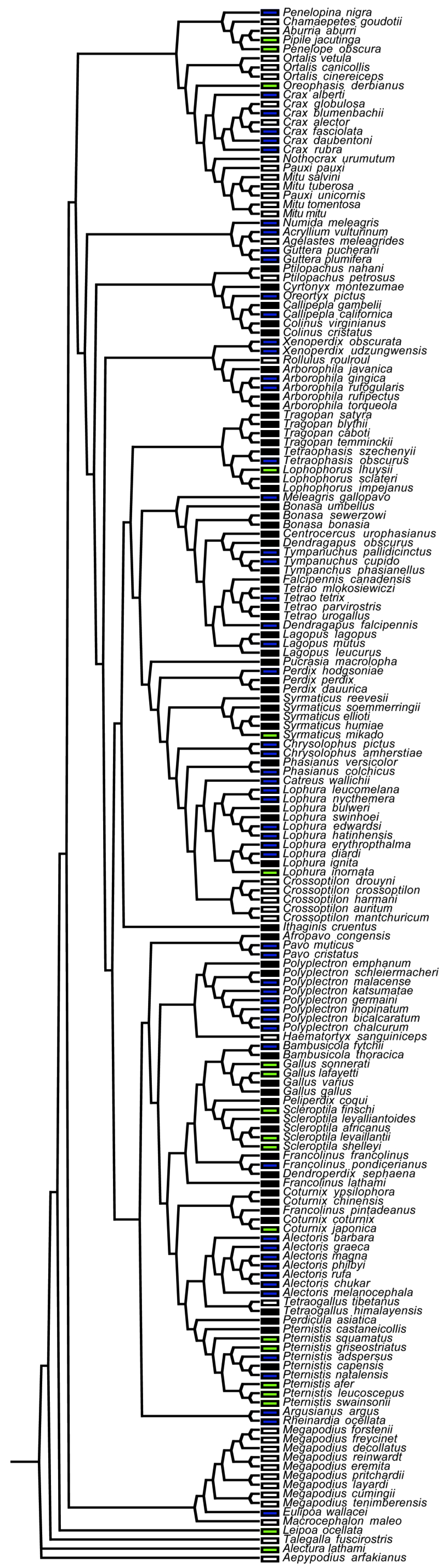
**

**
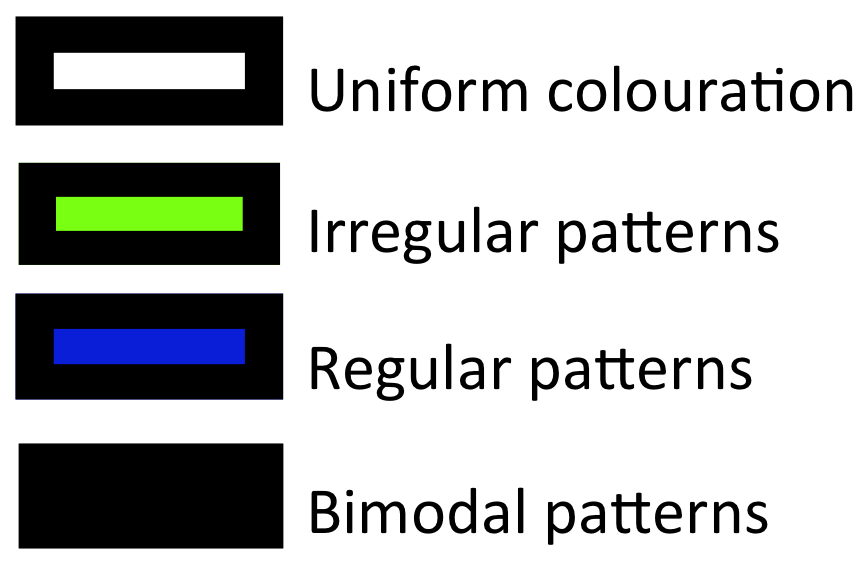
**

**Fig. S2.** The evolution of singular and bimodal

patterns in Galliformes. Both singular and

bimodal patterning is found in extant species.

Table. S1. A representative sample of literature examining pattern type and pattern function in different contexts (stationary or moving), in birds, mammals, fish, reptiles, insects, cephalopods, crustaceans, and amphibians. Literature is categorized as 1) Hypotheses (theoretical hypotheses), 2) Experimental (direct empirical evidence), 3) Correlational/observational, 4) Comparative phylogenetic analyses, and 5) Methodological (proposing and/or testing new methods). Camouflage mechanisms cover the following: Stationary camouflage (Background matching [BM], disruptive camouflage [DC]) and motion camouflage (motion dazzle [MD]), flicker-fusion [F-F]).

| **Pattern type** | **Behavioural context** | **Camouflage** | **Communication** | **Camouflage mechanism** | **Prey or signaller**  **(where tested)** | **Predator or signal receiver (where tested)** | | **References** | **Type of literature/ evidence** |
| --- | --- | --- | --- | --- | --- | --- | --- | --- | --- |
| **Irregular** | Stationary | X |  | BM | N/A | N/A | | ([Poulton, 1890](#_ENREF_69)) | Hypotheses |
|  | Stationary | X |  | BM and DC | N/A | N/A | | ([Thayer, 1909](#_ENREF_88)) | Hypotheses |
|  | Stationary | X |  | DC | N/A | N/A | | ([Cott, 1940](#_ENREF_18)) | Hypotheses |
|  | Stationary | X |  | BM and DC | Juvenile cuttlefish  (*Sepia officinalis*) | N/A | | ([Hanlon & Messenger, 1988](#_ENREF_35)) | Experimental |
|  | Stationary | X |  | DC | Frogs (*Limnodynastes tasmaniensis*) | Garter snake (*Thamnophis sirtalis*) | | ([Osorio & Srinivasan, 1991](#_ENREF_65)) | Experimental |
|  | Stationary | X |  | BM | *Aythya* and *Somateria* ducks | N/A | | ([Hohman et al., 1992](#_ENREF_37)) | Hypotheses |
|  | Stationary | X |  | DC | Marine isopod *Idotea baltica* (white-spotted phenotype *albafusca*) | N/A | | ([Merilaita, 1998](#_ENREF_56)) | Experimental |
|  | Stationary | X |  | BM | Artificial paper moths | Captive trained birds | | ([Merilaita et al., 2001](#_ENREF_59)) | Experimental |
| **Irregular** | Stationary  Stationary | X  X |  | BM and DC  BM and DC | Cuttlefish (*Sepia officinalis*)  Cuttlefish (*Sepia officinalis*) | Fish predators  Fish predators  (Akkaynak et al., 2013; Chiao et al., 2013; Hanlon et al., 2013) | | E.g. ([Chiao & Hanlon, 2001](#_ENREF_14); [Barbosa et al., 2004](#_ENREF_4); [Chiao et al., 2005](#_ENREF_15); [Barbosa et al., 2007](#_ENREF_6); [Chiao et al., 2007](#_ENREF_13); [Mäthger et al., 2007](#_ENREF_54); [Shohet et al., 2007](#_ENREF_76); [Barbosa et al., 2008](#_ENREF_5); [Mäthger et al., 2008](#_ENREF_55); [Chiao et al., 2010](#_ENREF_12); [Chiao et al., 2011](#_ENREF_17); [Akkaynak et al., 2013](#_ENREF_1); [Chiao et al., 2013](#_ENREF_16); [Hanlon et al., 2013](#_ENREF_34))  S | Experimental  Experimental |
|  | Stationary | X |  | BM | Artificial paper moths | Wild birds | | ([Cuthill et al., 2005](#_ENREF_19)) | Experimental |
|  | Stationary | X |  | BM and DC | Artificial paper moths | Captive trained birds | | ([Merilaita & Lind, 2005](#_ENREF_58)) | Experimental |
|  | Stationary | X |  | DC | Artificial paper moths | Wild birds | | ([Stevens & Cuthill, 2006](#_ENREF_79)) | Experimental |
|  | Stationary | X |  | DC | Artificial paper moths | Wild birds | | ([Stevens et al., 2006](#_ENREF_80)) | Experimental |
| **Irregular** | Stationary | X |  | BM and DC | Artificial paper moths | Wild birds | | ([Cuthill et al., 2006](#_ENREF_20)) | Experimental |
|  | Stationary | X |  | BM and DC | Artificial paper moths (modelled on *Thyatira batis*) | Wild birds | | ([Schaefer & Stobbe, 2006](#_ENREF_73)) | Experimental |
|  | Stationary | X |  | BM | Fiddler crabs (*Uca vomeris*) | Wild and dummy birds | | ([Hemmi et al., 2006](#_ENREF_36)) | Experimental |
|  | Stationary | X |  | DC | Computer-generated moth images | Humans | | ([Fraser et al., 2007](#_ENREF_28)) | Experimental |
|  | Stationary | X |  | DC (surface disruption) | Artificial paper moths | Wild birds | | ([Stevens et al., 2009](#_ENREF_82)) | Experimental |
|  | Moving and stationary | X |  | DC/MD | Cuttlefish (*Sepia officinalis*) | N/A | | ([Zylinski et al., 2009](#_ENREF_92)) | Experimental |
|  | Stationary | X |  | BM and DC | Artificial paper moths | Captive trained birds | | ([Dimitrova & Merilaita, 2010](#_ENREF_22)) | Experimental |
|  | Stationary | X |  | BM | Octopuses (*Octopus cyanea* & *O.vulgaris*) | N/A | | ([Josef et al., 2012](#_ENREF_41)) | Experimental |
|  | Stationary | X |  | DC | Computer-generated moth images | Humans | | ([Troscianko et al., 2013](#_ENREF_89)) | Experimental |
|  | Stationary | X |  | BM and DC | Computer-generated moth images | Humans | | [Webster et al., 2013](#_ENREF_83)) | Experimental |
|  | Moving and stationary | X |  | BM and DC | Computer-generated stimuli | Humans | | ([Hall et al., 2013](#_ENREF_33)) | Experimental |
| **Irregular** | Stationary | X |  | BM | Japanese quail (*Coturnix japonica*) | N/A | | ([Lovell et al., 2013](#_ENREF_51)) | Experimental |
|  | Stationary | X |  | BM and DC | Moths (*Jankowskia fuscaria*) | N/A | | ([Kang et al., 2012](#_ENREF_42), [2013a](#_ENREF_43), [2013b](#_ENREF_44); [Kang et al., 2014](#_ENREF_45)) | Experimental |
|  | N/A |  | X | N/A | Red-legged partridge (*Alectoris rufa*) | Red-legged partridge (*Alectoris rufa*) | | ([Pérez-Rodríguez et al., 2013](#_ENREF_66)) | Experimental |
|  | Stationary | X |  | BM | Artificial paper moths | Captive trained birds | | ([DImitrova & Merilaita, 2014](#_ENREF_24)) | Experimental |
|  | Stationary | X |  | BM | Artificial paper moths | Captive trained birds | | ([Merilaita & DImitrova, 2014](#_ENREF_57)) | Experimental |
| **Irregular**  **TOTAL** | **Stationary = 42**  **Moving & stationary = 2** | **44** | **1** | **BM = 35**  **DC = 33**  **MD = 1** | **9 species: birds, insects, cephalopods, crustaceans, amphibians** | **4 groups: birds, fish, snakes, humans** | | **Ca. 45+** | **Experimental = 41/45 (91%)**  **Hypotheses = 4/45 (8%)** |
|  |  |  |  |  |  |  | |  |  |
| **Pattern type** | **Behavioural context** | **Camouflage** | **Communication** | **Camouflage mechanism** | **Prey or signaller**  **(where tested)** | **Predator or signal receiver (where tested)** | | **References** | **Type of literature/ evidence** |
| **Regular** | Moving | X |  | F-F | Brightly-coloured snakes, e.g. coral snakes | N/A | | ([Pough, 1976](#_ENREF_68)) | Hypotheses |
|  | Moving | X |  | F-F | North American snakes (132 spp.) | N/A | | ([Jackson et al., 1976](#_ENREF_40)) | Correlational/ observational |
| **Regular** | Moving | X |  | F-F | Guppies (*Poecilia reticulata*) | N/A | | ([Endler, 1980](#_ENREF_26)) | Experimental |
|  | Moving | X |  | MD and F-F | Garter snakes (*Thamnophis ordinoides*) | N/A | | ([Brodie, 1989](#_ENREF_8), [1992](#_ENREF_9), [1993](#_ENREF_10)) *but see* [Allen et al. 2013](#_ENREF_2)) | Correlational/ observational |
|  | N/A | X |  | BM | Tiger (*Panthera tigris*) | N/A | | ([Godfrey et al., 1987](#_ENREF_32)) | Experimental |
|  | N/A |  | X | N/A | Peafowl (*Pavo cristatus*) | Peafowl (*Pavo cristatus*) | | ([Petrie et al., 1991](#_ENREF_67)) | Correlational/ observational |
|  | N/A | X |  | BM | Zebra, tigers | N/A | | ([Kiltie et al., 1994](#_ENREF_48)) | Methodological |
|  | Moving | X |  | F-F | Vipera snakes | N/A | | ([Shine & Madsen, 1994](#_ENREF_75)) *but see* Allen et al. (2013) | Hypotheses |
|  | N/A |  | X | N/A | Zebra finches (*Taeniopygia guttata*) | Zebra finches (*Taeniopygia guttata*) | | ([Swaddle & Cuthill, 1994](#_ENREF_87)) | Experimental |
|  | Moving | X |  | F-F | Adders (*Vipera berus*) | N/A | | ([Lindell & Forsman, 1996](#_ENREF_50)) | Correlational/ observational |
|  | N/A |  | X | N/A | Mallard ducks (*Anas platyrhynchos*) | Mallard ducks (*Anas platyrhynchos*) | | ([Omland, 1996](#_ENREF_63)) | Correlational/ observational |
|  | N/A | X |  | BM | Mammalian carnivores | N/A | ([Ortolani, 1999](#_ENREF_64)) | | Comparative phylogenetic |
| **Regular** | N/A |  | X | N/A | Barn owls  (*Tyto alba*) | Barn owls  (*Tyto alba*) | ([Roulin, 1999](#_ENREF_70)) | | Experimental |
|  | N/A | X |  | N/A | Artiodactyls | N/A | ([Stoner et al., 2003](#_ENREF_85)) | | Comparative phylogenetic |
|  | N/A |  | X | N/A | N/A | N/A | ([Kenward et al., 2004](#_ENREF_47)) | | Comparative phylogenetic |
|  | Moving and stationary | X | X | Private UV signals | Damselfish (*Pomacentrus amboinensis*) | N/A | ([Siebeck, 2004](#_ENREF_78)) | | Experimental |
|  | Stationary | X |  | DC | Clay models of adders  (*Vipera latastei gaditana*) | Wild birds | ([Niskanen & Mappes, 2005](#_ENREF_61)) | | Experimental |
|  | N/A |  | X | N/A | Red-legged partridge  (*Alectoris rufa*) | Red-legged partridge (*Alectoris rufa*) | ([Bortolotti et al., 2006](#_ENREF_7)) | | Correlational/ observational |
|  | Moving | X |  | MD | Cuttlefish  (*Sepia officinalis*) | N/A | ([Shohet et al., 2006](#_ENREF_77)) | | Experimental |
|  | Moving | X |  | MD and F-F | Computer-generated moving stimuli | Humans | ([Stevens et al., 2008](#_ENREF_83)) | | Experimental |
|  | Stationary | X |  | DC | Artificial paper butterflies (modelled on *Limenitis camilla*) | Wild birds | ([Stobbe & Schaefer, 2008](#_ENREF_84)) | | Experimental |
|  | Stationary | X |  | Coincident disruptive camouflage | Artificial paper moths/computer-generated stimuli | Wild birds/humans | ([Cuthill & Székely, 2009](#_ENREF_21)) | | Experimental |
|  | N/A |  | X | N/A | N/A | N/A | ([Gluckman & Cardoso, 2009](#_ENREF_30)) | | Methodological and Experimental |
| **Regular** | N/A |  | X | N/A | Barn owls  (*Tyto alba*) | Barn owls  (*Tyto alba*) | ([Roulin et al., 2010](#_ENREF_71)) | | Correlational/ observational, Experimental and Comparative phylogenetic |
|  | N/A | X |  | BM | Felidae | N/A | ([Allen et al., 2011](#_ENREF_3)) | | Comparative phylogenetic |
|  | N/A |  | X | N/A | Barred buttonquails (*Turnix suscitator*) | Barred buttonquails (*Turnix suscitator*) | ([Muck & Goymann, 2011](#_ENREF_60)) | | Correlational/ observational |
|  | Moving | X |  | MD and F-F | Computer-generated moving stimuli | Humans | ([Scott-Samuel et al., 2011](#_ENREF_74)) | | Experimental |
|  | Stationary | X |  | BM | The least killfish (*Heterandria formosa*) | Predatory fish | ([Kjernsmo & Merilaita, 2012](#_ENREF_49)) | | Experimental |
|  | Moving | X |  | MD | Computer-generated moving stimuli | Humans | ([von Helversen et al., 2013](#_ENREF_90)) | | Experimental |
|  | Moving | X |  | MD | Computer-generated moving stimuli | Locusts (*Schistocerca gregaria*) | ([Santer, 2013](#_ENREF_72)) | | Experimental |
|  | Moving | X |  | MD | Computer-generated moving stimuli | Motion detection algorithm | ([How & Zanker, 2013](#_ENREF_38)) | | Experimental |
| **Regular**  **TOTAL** | **Stationary = 4**  **Moving = 14**  **Moving & stationary = 1** | **24** | **10** | **F-F = 8**  **MD = 7**  **BM = 5**  **DC = 3**  **Private signals = 1** | **Ca. 13 species: birds, reptiles, cephalopods, fish, mammals** | **4 groups: fish, insects, birds, humans** | **Ca. 33+** | | **Experimental = 17/33 (52%)**  **Correlational comparative = 14/33 (42%)**  **Hypotheses = 6%** |
| **Irregular and regular** | N/A | X |  | BM | Juvenile plaice (*Pleuronectes platessa*) | N/A | ([Kelman et al., 2006](#_ENREF_46)) | | Experimental |
|  | Moving and stationary | X |  | MD, F-F, BM, DC | Computer-generated moving and static stimuli | Humans | ([Stevens et al., 2011](#_ENREF_81)) | | Experimental |
|  | Stationary | X |  | BM | Artificial paper moths | Captive trained birds | ([Dimitrova & Merilaita, 2012](#_ENREF_23)) | | Experimental |
|  | N/A | X |  | BM and DC | *Galaxias nebula* fish (Galaxiidae) | N/A | ([Magellan & Swartz, 2013](#_ENREF_52)) | | Experimental |
|  | Moving | X |  | MD, F-F, BM, distractive markings | Computer-generated moving stimuli | Humans | ([Hughes et al., 2014](#_ENREF_39)) | | Experimental |
| **Irregular and regular**  **TOTAL** | **Stationary = 2**  **Moving & stationary = 1** | **5** | **0** | **BM = 5**  **DC = 2**  **MD, F-F = 3** | **2 species (fish)** | **2 groups (birds and humans)** | **Ca. 5+** | | **Experimental = 5/5 (100%)** |
|  |  |  |  |  |  |  | |  |  |
| **Pattern type** | **Behavioural context** | **Camouflage** | **Communication** | **Camouflage mechanism** | **Prey or signaller**  **(where tested)** | **Predator or signal receiver (where tested)** | | **References** | **Type of literature/ evidence** |
| **Bimodal** | Moving and stationary | X | X | Camouflage vs. signal efficacy | Guppies (*Poecilia reticulata*) | Various aquatic predators | ([Endler, 1978](#_ENREF_25)) | | Hypotheses and Experimental |
|  | Moving and stationary | X | X | Predator avoidance vs. signal efficacy | N/A | N/A | ([Endler, 1987](#_ENREF_27)) | | Experimental |
|  | Moving and stationary | X | X | Distance-dependence | Reef fish (e.g. *Pygoplites diacanthus*) | Predatory fish | ([Marshall, 2000](#_ENREF_53)) | | Experimental |
| **Bimodal** | Stationary | X | X | Signal partitioning | Australian agamid lizards (*Agamidae*) | N/A | ([Stuart-Fox & Ord, 2004](#_ENREF_86)) | | Comparative phylogenetic |
|  | N/A | X | X | Signal partitioning | *Bicyclus* butterflies | N/A | ([Oliver et al., 2009](#_ENREF_62)) | | Comparative phylogenetic |
|  | N/A | X | X | Signal partitioning | Birds | N/A | ([Gluckman & Cardoso, 2010](#_ENREF_31)) | | Comparative phylogenetic |
|  | Stationary | X | X | Background-matching vs. conspicuous signalling | Giant cuttlefish (*Sepia apama*) | N/A | ([Zylinski et al., 2011](#_ENREF_91)) | | Experimental |
|  | N/A | X | X | Predator avoidance vs. sexual dichromatism | Australian dragon lizards (*Agamidae*) | N/A | ([Chen et al., 2012](#_ENREF_11)) | | Comparative phylogenetic |
|  | Stationary | X | X | Signal partitioning | Australian mallee dragon lizards (*Ctenophorus fordi*) | Avian predators | ([Garcia et al., 2013](#_ENREF_29)) | | Experimental |
| **Bimodal**  **TOTAL** | **Stationary = 3**  **Moving & stationary = 3** | **9** | **9** | **Multiple function = 5**  **Signal partitioning=4** | **Ca. 4+ species: reptiles, cuttlefish, birds, insects, fish** | **2 groups (fish, birds)** | **Ca. 9+** | | **Experimental= 5/9 (55%)**  **Comparative = 4/9 (45%)** |

**Table S2** Character states across Anseriformes in taxonomic order

| **Species** | **Class** |
| --- | --- |
| *Anas aucklandica* | Regular |
| *Anas chlorotis* | Regular |
| *Anas bernieri* | Irregular |
| *Anas castanea* | Irregular |
| *Anas gibberifrons* | Regular |
| *Anas f. flavirostris* | Regular |
| *Anas f. oxyptera* | Bimodal |
| *Anas carolinensis* | Irregular |
| *Anas crecca* | Regular |
| *Anas acuta* | Bimodal |
| *Anas georgica* | Uniform |
| *Anas erythrorhyncha* | Regular |
| *Anas bahamensis* | Regular |
| *Anas capensis* | Regular |
| *Anas diazi* | Regular |
| *Anas rubripes* | Uniform |
| *Anas fulvigula* | Bimodal |
| *Anas platyrhynchos* | Bimodal |
| *Anas poecilorhyncha* | Regular |
| *Anas luzonica* | Uniform |
| *Anas laysanensis* | Bimodal |
| *Anas superciliosa* | Uniform |
| *Anas melleri* | Irregular |
| *Anas undulata* | Bimodal |
| *Anas sparsa* | Irregular |
| *Anas americana* | Regular |
| *Anas sibilatrix* | Regular |
| *Anas penelope* | Regular |
| *Anas falcata* | Regular |
| *Anas strepera* | Bimodal |
| *Anas clypeata* | Regular |
| *Anas rhynchotis* | Regular |
| *Anas smithii* | Bimodal |
| *Anas c. cyanoptera* | Uniform |
| *Anas discors* | Regular |
| *Anas c. septentrionalium* | Irregular |
| *Anas platalea* | Regular |
| *Anas v. puna* | Bimodal |
| *Anas versicolor* | Regular |
| *Anas hottentota* | Regular |
| *Anas querquedula* | Regular |
| *Anas formosa* | Regular |
| *Amazonetta brasiliensis* | Regular |
| *Speculanas specularis* | Irregular |
| *Lophonetta specularoides* | Irregular |
| *Tachyeres pteneres* | Regular |
| *Aythya affinis* | Regular |
| *Aythya marila* | Regular |
| *Aythya fuligula* | Uniform |
| *Aythya americana* | Regular |
| *Aythya ferina* | Regular |
| *Aythya australis* | Uniform |
| *Aythya nyroca* | Uniform |
| *Netta peposaca* | Regular |
| *Netta rufina* | Uniform |
| *Asarcornis scutulata* | Irregular |
| *Marmaronetta angustirostris* | Regular |
| *Cyanochen cyanoptera* | Irregular |
| *Pteronetta hartlaubii* | Uniform |
| *Chenonetta jubata* | Bimodal |
| *Sarkidiornis melanotos* | Regular |
| *Callonetta leucophrys* | Regular |
| *Tadorna ferruginea* | Uniform |
| *Tadorna tadornoides* | Regular |
| *Tadorna cana* | Uniform |
| *Tadorna tadorna* | Uniform |
| *Alopochen aegyptiacus* | Uniform |
| *Tadorna radjah* | Uniform |
| *Chloephaga picta* | Regular |
| *Chloephaga poliocephala* | Regular |
| *Aix galericulata* | Bimodal |
| *Aix sponsa* | Bimodal |
| *Cairina moschata* | Uniform |
| *Mergus merganser* | Uniform |
| *Mergus serrator* | Bimodal |
| *Lophodytes cucullatus* | Regular |
| *Mergellus albellus* | Uniform |
| *Bucephala clangula* | Uniform |
| *Bucephala islandica* | Uniform |
| *Bucephala albeola* | Uniform |
| *Melanitta nigra* | Uniform |
| *Melanitta perspicillata* | Uniform |
| *Somateria mollissima* | Regular |
| *Somateria spectabilis* | Regular |
| *Clangula hyemalis* | Uniform |
| *Anser albifrons* | Bimodal |
| *Anser erythropus* | Uniform |
| *Anser fabalis* | Regular |
| *Anser anser* | Bimodal |
| *Anser cygnoides* | Regular |
| *Anser brachyrhynchus* | Regular |
| *Anser indicus* | Uniform |
| *Anser rossii* | Uniform |
| *Anser canagicus* | Regular |
| *Branta canadensis* | Uniform |
| *Branta sandvicensis* | Bimodal |
| *Branta leucopsis* | Regular |
| *Branta ruficollis* | Uniform |
| *Branta bernicla* | Uniform |
| *Cygnus atratus* | Uniform |
| *Cygnus olor* | Uniform |
| *Cygnus columbianus* | Uniform |
| *Cygnus cygnus* | Uniform |
| *Cygnus melanocorypha* | Uniform |
| *Coscoroba coscoroba* | Uniform |
| *Cereopsis novaehollandiae* | Regular |
| *Malacorhynchus membranaceus* | Regular |
| *Oxyura australis* | Regular |
| *Oxyura maccoa* | Uniform |
| *Oxyura jamaicensis* | Uniform |
| *Oxyura vittata* | Uniform |
| *Oxyura leucocephala* | Regular |
| *Nomonyx dominicus* | Bimodal |
| *Biziura lobata* | Regular |
| *Dendrocygna bicolor* | Bimodal |
| *Dendrocygna javanica* | Regular |
| *Dendrocygna eytoni* | Bimodal |
| *Dendrocygna viduata* | Regular |

**Table S3** Character states across Galliformes in taxonomic order

| **Species** | **Class** |
| --- | --- |
| *Megapodius forstenii* | Uniform |
| *Megapodius freycinet* | Uniform |
| *Megapodius decollatus* | Uniform |
| *Megapodius reinwardt* | Uniform |
| *Megapodius eremita* | Uniform |
| *Megapodius pritchardii* | Uniform |
| *Megapodius layardi* | Uniform |
| *Megapodius cumingii* | Uniform |
| *Megapodius tenimberensis* | Uniform |
| *Eulipoa wallacei* | Regular |
| *Macrocephalon maleo* | Uniform |
| *Alectura lathami* | Irregular |
| *Aepypodius arfakianus* | Uniform |
| *Talegalla fuscirostris* | Uniform |
| *Leipoa ocellata* | Irregular |
| *Crax globulosa* | Uniform |
| *Crax blumenbachii* | Regular |
| *Crax alector* | Uniform |
| *Crax fasciolata* | Regular |
| *Crax daubentoni* | Regular |
| *Crax rubra* | Regular |
| *Crax alberti* | Regular |
| *Mitu tomentosa* | Uniform |
| *Mitu mitu* | Uniform |
| *Mitu salvini* | Uniform |
| *Mitu tuberosa* | Uniform |
| *Pauxi unicornis* | Uniform |
| *Pauxi pauxi* | Uniform |
| *Nothocrax urumutum* | Uniform |
| *Oreophasis derbianus* | Irregular |
| *Ortalis vetula* | Uniform |
| *Ortalis canicollis* | Uniform |
| *Ortalis cinereiceps* | Uniform |
| *Aburria aburri* | Uniform |
| *Pipile jacutinga* | Irregular |
| *Penelope obscura* | Irregular |
| *Chamaepetes goudotii* | Uniform |
| *Penelopina nigra* | Regular |
| *Guttera pucherani* | Regular |
| *Guttera plumifera* | Regular |
| *Acryllium vulturinum* | Regular |
| *Agelastes meleagrides* | Uniform |
| *Numida meleagris* | Regular |
| *Ptilopachus nahani* | Bimodal |
| *Ptilopachus petrosus* | Uniform |
| *Colinus virginianus* | Bimodal |
| *Colinus cristatus* | Bimodal |
| *Callipepla gambelii* | Bimodal |
| *Callipepla californica* | Regular |
| *Oreortyx pictus* | Regular |
| *Cyrtonyx montezumae* | Bimodal |
| *Arborophila gingica* | Regular |
| *Arborophila rufogularis* | Regular |
| *Arborophila rufipectus* | Bimodal |
| *Arborophila torqueola* | Bimodal |
| *Arborophila javanica* | Bimodal |
| *Rollulus roulroul* | Uniform |
| *Xenoperdix udzungwensis* | Regular |
| *Xenoperdix obscurata* | Regular |
| *Lophura diardi* | Regular |
| *Lophura ignita* | Bimodal |
| *Lophura erythropthalma* | Regular |
| *Lophura inornata* | Irregular |
| *Lophura edwardsi* | Regular |
| *Lophura hatinhensis* | Regular |
| *Lophura swinhoei* | Bimodal |
| *Lophura bulweri* | Bimodal |
| *Lophura leucomelana* | Regular |
| *Lophura nycthemera* | Regular |
| *Catreus wallichii* | Regular |
| *Crossoptilon crossoptilon* | Uniform |
| *Crossoptilon harmani* | Uniform |
| *Crossoptilon drouyni* | Uniform |
| *Crossoptilon auritum* | Uniform |
| *Crossoptilon mantchuricum* | Uniform |
| *Phasianus versicolor* | Bimodal |
| *Phasianus colchicus* | Regular |
| *Chrysolophus pictus* | Regular |
| *Chrysolophus amherstiae* | Regular |
| *Syrmaticus ellioti* | Bimodal |
| *Syrmaticus humiae* | Bimodal |
| *Syrmaticus mikado* | Irregular |
| *Syrmaticus soemmerringii* | Bimodal |
| *Syrmaticus reevesii* | Bimodal |
| *Perdix hodgsoniae* | Regular |
| *Perdix perdix* | Bimodal |
| *Perdix dauurica* | Bimodal |
| *Pucrasia macrolopha* | Bimodal |
| *Tympanuchus cupido* | Regular |
| *Tympanchus phasianellus* | Bimodal |
| *Tympanuchus pallidicinctus* | Regular |
| *Dendragapus obscurus* | Bimodal |
| *Centrocercus urophasianus* | Bimodal |
| *Tetrao mlokosiewiczi* | Bimodal |
| *Tetrao tetrix* | Regular |
| *Tetrao parvirostris* | Bimodal |
| *Tetrao urogallus* | Bimodal |
| *Falcipennis canadensis* | Bimodal |
| *Dendragapus falcipennis* | Regular |
| *Lagopus lagopus* | Bimodal |
| *Lagopus mutus* | Regular |
| *Lagopus leucurus* | Bimodal |
| *Bonasa sewerzowi* | Bimodal |
| *Bonasa bonasia* | Bimodal |
| *Bonasa umbellus* | Bimodal |
| *Meleagris gallopavo* | Regular |
| *Tragopan satyra* | Bimodal |
| *Tragopan blythii* | Bimodal |
| *Tragopan caboti* | Bimodal |
| *Tragopan temminckii* | Bimodal |
| *Tetraophasis szechenyii* | Bimodal |
| *Tetraophasis obscurus* | Regular |
| *Lophophorus lhuysii* | Irregular |
| *Lophophorus sclateri* | Bimodal |
| *Lophophorus impejanus* | Bimodal |
| *Ithaginis cruentus* | Bimodal |
| *Bambusicola cruentus* | Regular |
| *Bambusicola thoracica* | Bimodal |
| *Gallus sonnerati* | Irregular |
| *Gallus lafayetti* | Irregular |
| *Gallus varius* | Bimodal |
| *Gallus gallus* | Bimodal |
| *Peliperdix coqui* | Bimodal |
| *Scleroptila finschi* | Irregular |
| *Scleroptila levalliantoides* | Bimodal |
| *Scleroptila africanus* | Bimodal |
| *Scleroptila levaillantii* | Irregular |
| *Scleroptila shelleyi* | Irregular |
| *Francolinus francolinus* | Bimodal |
| *Francolinus pondicerianus* | Regular |
| *Dendroperdix sephaena* | Bimodal |
| *Francolinus lathami* | Bimodal |
| *Alectoris magna* | Regular |
| *Alectoris philbyi* | Regular |
| *Alectoris rufa* | Regular |
| *Alectoris chukar* | Regular |
| *Alectoris graeca* | Regular |
| *Alectoris melanocephala* | Regular |
| *Alectoris barbara* | Regular |
| *Tetraogallus tibetanus* | Uniform |
| *Tetraogallus himalayensis* | Bimodal |
| *Pternistis afer* | Irregular |
| *Pternistis leucoscepus* | Irregular |
| *Pternistis swainsonii* | Irregular |
| *Pternistis adspersus* | Regular |
| *Pternistis capensis* | Bimodal |
| *Pternistis natalensis* | Regular |
| *Pternistis griseostriatus* | Irregular |
| *Pternistis squamatus* | Irregular |
| *Pternistis castaneicollis* | Bimodal |
| *Perdicula asiatica* | Bimodal |
| *Coturnix ypsilophora* | Bimodal |
| *Coturnix chinensis* | Bimodal |
| *Francolinus pintadeanus* | Bimodal |
| *Coturnix coturnix* | Bimodal |
| *Coturnix japonica* | Irregular |
| *Polyplectron emphanum* | Bimodal |
| *Polyplectron schleiermacheri* | Bimodal |
| *Polyplectron malacense* | Regular |
| *Polyplectron katsumatae* | Regular |
| *Polyplectron germaini* | Regular |
| *Polyplectron inopinatum* | Regular |
| *Polyplectron bicalcaratum* | Regular |
| *Polyplectron chalcurum* | Regular |
| *Haematortyx sanguiniceps* | Uniform |
| *Afropavo congensis* | Bimodal |
| *Pavo muticus* | Regular |
| *Pavo cristatus* | Regular |
| *Argusianus argus* | Regular |
| *Rheinardia ocellata* | Regular |

**Table S4.** Calculation of prior odds of seeing any particular model of evolution on the basis of the number of transition parameters (*see* Currie *et al*. 2010 for a detailed description of the calculations used).

| **No. of Zeros (Z)** | **Binomial for Z** | **12 - Z** | **Bell number for 12 - Z** | **Binomial for Z * Bell number for 12 - Z** | **Bell + Total** | **Prior** |
| --- | --- | --- | --- | --- | --- | --- |
| 0 | 1 | 12 | 4213597 | 4213597 | 23430839 | 0.21926128 |
| 1 | 12 | 11 | 678570 | 8142840 | 24109409 | 0.02896055 |
| 2 | 66 | 10 | 115975 | 7654350 | 23546814 | 0.00494967 |
| 3 | 220 | 9 | 21147 | 4652340 | 23451986 | 0.00090253 |
| 4 | 495 | 8 | 4140 | 2049300 | 23434979 | 0.00017669 |
| 5 | 792 | 7 | 877 | 694584 | 23431716 | 0.00003743 |
| 6 | 924 | 6 | 203 | 187572 | 23431042 | 0.00000866 |
| 7 | 792 | 5 | 52 | 41184 | 23430891 | 0.00000222 |
| 8 | 495 | 4 | 15 | 7425 | 23430854 | 0.00000064 |
| 9 | 220 | 3 | 5 | 1100 | 23430844 | 0.00000021 |
| 10 | 66 | 2 | 2 | 132 | 23430841 | 0.00000009 |
| 11 | 12 | 1 | 1 | 12 | 23430840 | 0.00000004 |
|  |  |  | Total: | 23430839 |  |  |

**References**

Akkaynak, D., Allen, J. J., Mäthger, L. M., Chiao, C.-C., & Hanlon, R. T. (2013). Quantification of cuttlefish (Sepia officinalis) camouflage: a study of color and luminance using in situ spectrometry. *Journal of Comparative Physiology A, 199*(3), 211-225.

Allen, W. L., Baddeley, R., Scott-Samuel, N. E., & Cuthill, I. C. (2013). The evolution and function of pattern diversity in snakes. *Behavioral Ecology, 24*(5), 1237-1250.

Allen, W. L., Cuthill, I. C., Scott-Samuel, N. E., & Baddeley, R. (2011). Why the leopard got its spots: relating pattern development to ecology in felids. *Proceedings of the Royal Society, Series B, 278*(1710), 1373-1380.

Barbosa, A., Florio, C. F., Chiao, C.-C., & Hanlon, R. T. (2004). Visual background features that elicit mottled patterns in cuttlefish, *Sepia officinalis*. *The Biological Bulletin, 207*(2), 154.

Barbosa, A., Mäthger, L. M., Buresch, K. C., Kelly, J., Chubb, C., Chiao, C.-C., & Hanlon, R. T. (2008). Cuttlefish camouflage: The effects of substrate contrast and size in evoking uniform, mottle or disruptive body patterns. *Vision Research, 48*(10), 1242-1253.

Barbosa, A., Mäthger, L. M., Chubb, C., Florio, C. F., Chiao, C.-C., & Hanlon, R. T. (2007). Disruptive coloration in cuttlefish: a visual perception mechanism that regulates ontogenetic adjustment of skin patterning. *Journal of Experimental Biology, 210*, 1139-1147.

Bortolotti, G. R., Blas, J., Negro, J. J., & Tella, J. L. (2006). A complex plumage pattern as an honest social signal. *Animal Behaviour, 72*(2), 423-430.

Brodie, E. D. (1989). Genetic correlations between morphology and antipredator behaviour in natural populations of the garter snake *Thamnophis ordinoides*. *Nature, 342*, 542-543.

Brodie, E. D. (1992). Correlational selection for color pattern and antipredator behavior in the garter snake *Thamnophis ordinoides*. *Evolution, 46*(5), 1284-1298.

Brodie, E. D. (1993). Differential avoidance of coral snake banded patterns by free-ranging avian predators in Costa Rica. *Evolution, 47*(1), 227-235.

Chen, I.-P., Stuart-Fox, D. M., Hugall, A. F., & Symonds, M. R. E. (2012). Sexual selection and the evolution of complex color patterns in dragon lizards. *Evolution, 66*(11), 3605-3614.

Chiao, C.-C., Chubb, C., Buresch, K. C., Barbosa, A., Allen, J. J., Mäthger, L. M., & Hanlon, R. T. (2010). Mottle camouflage patterns in cuttlefish: quantitative characterization and visual background stimuli that evoke them. *Journal of Experimental Biology, 213*, 187-199.

Chiao, C.-C., Chubb, C., & Hanlon, R. T. (2007). Interactive effects of size, contrast, intensity and configuration of background objects in evoking disruptive camouflage in cuttlefish. *Vision Research, 47*(16), 2223-2235.

Chiao, C.-C., & Hanlon, R. T. (2001). Cuttlefish camouflage: visual perception of size, contrast and number of white squares on artificial checkerboard substrata initiates disruptive coloration. *Journal of Experimental Biology, 204*, 2119-2125.

Chiao, C.-C., Kelman, E. J., & Hanlon, R. T. (2005). Disruptive body patterning of cuttlefish (*Sepia officinalis*) requies visual information regarding edges and contrast of objects in natural substrate backgrounds. *The Biological Bulletin, 208*(1), 7-11.

Chiao, C.-C., Ulmer, K. M., Siemann, L. A., Buresch, K. C., Chubb, C., & Hanlon, R. T. (2013). How visual edge features influence cuttlefish camouflage patterning. *Vision Research, 83*, 40-47.

Chiao, C.-C., Wickiser, J. K., Allen, J. J., Genter, B., & Hanlon, R. T. (2011). Hyperspectral imaging of cuttlefish camouflage indicates good color match in the eyes of fish predators. *Proceedings of the National Academy of Sciences USA, 108*(22), 9148-9153.

Cott, H. B. (1940). *Adaptive coloration in animals*. London: Methuen & Co. Ltd.

Cuthill, I. C., Stevens, M., Sheppard, J., Maddocks, T., Párraga, C. A., & Troscianko, T. S. (2005). Disruptive coloration and background pattern matching. *Nature, 434*, 72-74.

Cuthill, I. C., Stevens, M., Windsor, A. M. M., & Walker, H. J. (2006). The effects of pattern symmetry on detection of disruptive and background-matching coloration. *Behavioral Ecology, 17*(5), 828-832.

Cuthill, I. C., & Székely, A. (2009). Coincident disruptive coloration. *Philosophical Transactions of the Royal Society B, 364*(1516), 489-496.

Dimitrova, M., & Merilaita, S. (2010). Prey concealment: visual background complexity and prey contrast distribution. *Behavioral Ecology, 21*(1), 176-181.

Dimitrova, M., & Merilaita, S. (2012). Prey pattern regularity and background complexity affect detectability of background-matching prey. *Behavioral Ecology, 23*(2), 384-390.

DImitrova, M., & Merilaita, S. (2014). Hide and seek: properties of prey and background patterns affect prey detection by blue tits. *Behavioral Ecology, 25*(2), 402-408.

Endler, J. A. (1978). A predator's view of animal color patterns. *Evolutionary Biology, 11*, 319-364.

Endler, J. A. (1980). Natural selection on color patterns in *Poecilia reticulata*. *Evolution, 34*(1), 76-91.

Endler, J. A. (1987). Predation, light intensity and courtship behavior in Poecilia reticulata (Pisces: Poeciliidae). *Animal Behaviour, 35*(5), 1376-1385.

Fraser, S., Callahan, A., Klassen, D., & Sherratt, T. N. (2007). Empirical tests of the role of disruptive coloration in reducing detectability. *Proceedings of the Royal Society, Series B, 274*(1615), 1325-1331.

Garcia, J. E., Rohr, D., & Dyer, A. G. (2013). Trade-off between camouflage and sexual dimorphism revealed by UV digital imaging: the case of Australian Mallee dragons (*Ctenophorus fordi*). *Journal of Experimental Biology, 216*, 4290-4298.

Gluckman, T.-L., & Cardoso, G. C. (2009). A method to quantify the regularity of barred plumage patterns. *Behavioral Ecology and Sociobiology, 63*(12), 1837-1844.

Gluckman, T.-L., & Cardoso, G. C. (2010). The dual function of barred plumage in birds: camouflage and communication. *Journal of Evolutionary Biology, 23*(11), 2501-2506.

Godfrey, D., Lythgoe, J. N., & Rumball, D. A. (1987). Zebra stripes and tiger stripes: the spatial frequency distribution of the pattern compared to that of the background is significant in display and crypsis. *Biological Journal of the Linnean Society, 32*(4), 427-433.

Hall, J. R., Cuthill, I. C., Baddeley, R., Shohet, A. J., & Scott-Samuel, N. E. (2013). Camouflage, detection and identification of moving targets. *Proceedings of the Royal Society, Series B, 280*(1758), 20130064.

Hanlon, R. T., Chiao, C.-C., Mäthger, L. M., & Marshall, J. (2013). A fish-eye view of cuttlefish camouflage using in situ spectrometry. *Biological Journal of the Linnean Society, 109*(3), 535-551.

Hanlon, R. T., & Messenger, J. B. (1988). Adaptive coloration in young cuttlefish (*Sepia officinalis L.*): the morphology and development of body patterns and their relation to behaviour. *Philosophical Transactions of the Royal Society B, 320*(1200), 437-487.

Hemmi, J. M., Marshall, J., Pix, W., Vorobyev, M., & Zeil, J. (2006). The variable colours of the fiddler crab Uca vomeris and their relation to background and predation. *Journal of Experimental Biology, 209*, 4140-4153.

Hohman, W. L., Ankney, C. D., & Gordon, D. H. (1992). Ecology and management of postbreeding waterfowl. In B. D. J. Batt, A. D. Afton, M. G. Anderson, C. D. Ankney, D. H. Johnson, J. A. Kadlec & G. L. Krapu (Eds.), *Ecology and management of breeding waterfowl* (pp. 128-189). Minneapolis: University of Minnesota Press.

How, M. J., & Zanker, J. M. (2013). Motion camouflage induced by zebra stripes. *Zoology, 117*(3), 163-170.

Hughes, A. E., Troscianko, J., & Stevens, M. (2014). Motion dazzle and the effects of target patterning on capture success. *BMC Evolutionary Biology, 14*, 201.

Jackson, J. F., Ingram, W., & Campbell, H. W. (1976). The dorsal pigmentation pattern of snakes as an antipredator strategy: A multivariate approach. *The American Naturalist, 110*(976), 1029-1053.

Josef, N., Amodio, P., Fiorito, G., & Shashar, N. (2012). Camouflaging in a complex environment-Octopuses use specific features of their surroundings for background matching. *PLoS ONE, 7*(5), e37579.

Kang, C.-K., Moon, J.-Y., Lee, S.-I., & Jablonski, P. G. (2012). Camouflage through an active choice of a resting spot and body orientation in moths. *Journal of Evolutionary Biology, 25*, 1695-1702.

Kang, C.-K., Moon, J.-Y., Lee, S.-I., & Jablonski, P. G. (2013a). Cryptically patterned moths perceive bark structure when choosing body orientations that match wing color pattern to the bark pattern. *PLoS ONE, 8*, e78117.

Kang, C.-K., Moon, J.-Y., Lee, S.-I., & Jablonski, P. G. (2013b). Moths on tree trunks seek out more cryptic positions when their current crypticity is low. *Animal Behaviour, 86*(3), 587-594.

Kang, C.-K., Stevens, M., Moon, J.-Y., Lee, S.-I., & Jablonski, P. G. (2014). Camouflage through behavior in moths: the role of background matching and disruptive coloration. *Behavioral Ecology*. doi: 10.1093/beheco/aru150

Kelman, E. J., Tiptus, P., & Osorio, D. (2006). Juvenile plaice (Pleuronectes platessa) produce camouflage by flexibly combining two separate patterns. *Journal of Experimental Biology, 209*, 3288-3292.

Kenward, B., Wachtmeister, C. A., Ghirlanda, S., & Enquist, M. (2004). Spots and stripes: the evolution of repetition in visual signal form. *Journal of Theoretical Biology, 230*(3), 407-419.

Kiltie, R. A., Fan, J., & Laine, A. F. (1994). A wavelet-based metric for visual texture discrimination with applications in evolutionary ecology. *Mathematical Biosciences, 126*(1), 21-39.

Kjernsmo, K., & Merilaita, S. (2012). Background choice as an anti-predator strategy: the roles of background matching and visual complexity in the habitat choice of the least killifish. *Proceedings of the Royal Society, Series B, 279*(1745), 4192-4198.

Lindell, L. E., & Forsman, A. (1996). Density effects and snake predation: prey limitation and reduced growth rate of adders at high density of conspecifics. *Canadian Journal of Zoology, 74*(6), 1000-1007.

Lovell, P. G., Ruxton, G. D., Langridge, K. V., & Spencer, K. A. (2013). Egg-laying substrate selection for optimal camouflage by quail. *Current Biology, 23*(3), 260-264.

Magellan, K., & Swartz, E. R. (2013). Crypsis in a heterogeneous environment: relationships between changeable polymorphic colour patterns and behaviour in a galaxiid fish. *Freshwater Biology, 58*(4), 793-799.

Marshall, J. (2000). Communication and camouflage with the same 'bright' colours in reef fishes. *Philosophical Transactions of the Royal Society B, 355*(1401), 1243-1248.

Mäthger, L. M., Chiao, C.-C., Barbosa, A., Buresch, K. C., Kaye, S., & Hanlon, R. T. (2007). Disruptive coloration elicited on controlled natural substrates in cuttlefish, *Sepia officinalis*. *Journal of Evolutionary Biology, 210*, 2657-2666.

Mäthger, L. M., Chiao, C.-C., Barbosa, A., & Hanlon, R. T. (2008). Color matching on natural substrates in cuttlefish, Sepia officinalis. *Journal of Comparative Physiology A, 194*(6), 577-585.

Merilaita, S. (1998). Crypsis through disruptive coloration in an isopod. *Proceedings of the Royal Society, Series B, 265*(1401), 1059-1064.

Merilaita, S., & DImitrova, M. (2014). Accuracy of background matching and prey detection: predation by blue tits indicates intense selection for highly matching prey colour pattern. *Functional Ecology*. doi: 10.1111/1365-2435.12248

Merilaita, S., & Lind, J. (2005). Background-matching and disruptive coloration, and the evolution of cryptic coloration. *Proceedings of the Royal Society, Series B, 272*(1563), 665-670.

Merilaita, S., Lyytinen, A., & Mappes, J. (2001). Selection for cryptic coloration in a visually heterogeneous habitat. *Proceedings of the Royal Society, Series B, 268*(1479), 1925-1929.

Muck, C., & Goymann, W. (2011). Throat patch size and darkness covaries with testosterone in females of a sex-role reversed species. *Behavioral Ecology, 22*(6), 1312-1319.

Niskanen, M., & Mappes, J. (2005). Significance of the dorsal zigzag pattern of *Vipera latastei gaditana* against avian predators. *Journal of Animal Ecology, 74*(6), 1091-1101.

Oliver, J. C., Robertson, K. A., & Monteiro, A. (2009). Accommodating natural and sexual selection in butterfly wing pattern evolution. *Proceedings of the Royal Society, Series B, 276*(1666), 2369-2375.

Omland, K. E. (1996). Female mallard mating preferences for multiple male ornaments. *Behavioral Ecology and Sociobiology, 39*(6), 353-360.

Ortolani, A. (1999). Spots, stripes, tail tips and dark eyes: Predicting the function of carnivore colour patterns using the comparative method. *Biological Journal of the Linnean Society, 67*(4), 433-476.

Osorio, D., & Srinivasan, M. V. (1991). Camouflage by edge enhancement in animal coloration patterns and its implications for visual mechanisms. *Proceedings of the Royal Society, Series B, 244*(1310), 81-85.

Pérez-Rodríguez, L., Jovani, R., & Mougeot, F. (2013). Fractal geometry of a complex plumage trait reveals bird's quality. *Proceedings of the Royal Society, Series B, 280*(1755), 20122783.

Petrie, M., Halliday, T., & Sanders, C. (1991). Peahens prefer peacocks with elaborate trains. *Animal Behaviour, 41*(2), 323-331.

Pough, F. H. (1976). Multiple cryptic effects of crossbanded and ringed patterns of snakes. *Copeia, 1976*(4), 834-836.

Poulton, E. B. (1890). The colours of animals: their meaning and use. Especially considered in the case of insects. *The International Scientific Series* (2nd ed.). London: Kegan Paul, Trench Trübner & Co. Ltd.

Roulin, A. (1999). Nonrandom pairing by male barn owls (*Tyto alba*) with respect to a female plumage trait. *Behavioral Ecology, 10*(6), 688-695.

Roulin, A., Altwegg, R., Jensen, H., Steinsland, I., & Schaub, M. (2010). Sex-dependent selection on an autosomal melanic female ornament promotes the evolution of sex ratio bias. *Ecology Letters, 13*(5), 616-626.

Santer, R. D. (2013). Motion dazzle: a locust's eye view. *Biology Letters, 9*(6), 20130811.

Schaefer, M. H., & Stobbe, N. (2006). Disruptive coloration provides camouflage independent of background matching. *Proceedings of the Royal Society, Series B, 273*(1600), 2427-2432.

Scott-Samuel, N. E., Baddeley, R., Palmer, C. E., & Cuthill, I. C. (2011). Dazzle camouflage affects speed perception. *PLoS ONE, 6*, e20233.

Shine, R., & Madsen, T. (1994). Sexual dichromatism in snakes of the genus Vipera: A review and a new evolutionary hypothesis. *Journal of Herpetology, 28*, 114-117.

Shohet, A. J., Baddeley, R., Anderson, J., & Osorio, D. (2007). Cuttlefish camouflage: a quantitative study of patterning. *Biological Journal of the Linnean Society, 92*(2), 335-345.

Shohet, A. J., Baddeley, R., Anderson, J. C., Kelman, E. J., & Osorio, D. (2006). Cuttlefish responses to visual orientation of substrates, water flow and a model of motion camouflage. *Journal of Experimental Biology, 209*, 4717-4723.

Siebeck, U. E. (2004). Communication in coral reef fish: the role of ultraviolet colour patterns in damselfish territorial behaviour. *Animal Behaviour, 68*(2), 273-282.

Stevens, M., & Cuthill, I. C. (2006). Disruptive coloration, crypsis and edge detection in early visual processing. *Proceedings of the Royal Society, Series B, 273*(1598), 2141-2147.

Stevens, M., Cuthill, I. C., Windsor, A. M. M., & Walker, H. J. (2006). Disruptive contrast in animal camouflage. *Proceedings of the Royal Society, Series B, 273*(1600), 2433-2438.

Stevens, M., Searle, W. T., Seymour, J., Marshall, K. L. A., & Ruxton, G. D. (2011). Motion dazzle and camouflage as distinct anti-predator defenses. *BMC Biology, 9*, 81.

Stevens, M., Winney, I. S., Cantor, A., & Graham, J. (2009). Outline and surface disruption in animal camouflage. *Proceedings of the Royal Society, Series B, 276*(1657), 781-786.

Stevens, M., Yule, D. H., & Ruxton, G. D. (2008). Dazzle coloration and prey movement. *Proceedings of the Royal Society, Series B, 275*(1651), 2639-2643.

Stobbe, N., & Schaefer, M. H. (2008). Enhancement of chromatic contrast increases predation risk for striped butterflies. *Proceedings of the Royal Society, Series B, 275*(1642), 1535-1541.

Stoner, C. J., Caro, T., & Graham, C. M. (2003). Ecological and behavioral correlates of coloration in artiodactyls: systematic analyses of conventional hypotheses. *Behavioral Ecology, 14*(6), 823-840.

Stuart-Fox, D. M., & Ord, T. J. (2004). Sexual selection, natural selection and the evolution of dimorphic coloration and ornamentation in agamid lizards. *Proceedings of the Royal Society, Series B, 271*(1554), 2249-2255.

Swaddle, J. P., & Cuthill, I. C. (1994). Female zebra finches prefer males with symmetric chest plumage. *Proceedings of the Royal Society, Series B, 258*(1353), 267-271.

Thayer, G. H. (1909). *Concealing-coloration in the animal kingdom: an exposition of the laws of disguise through color and pattern: being a summary of Abbott H. Thayer's discoveries.* New York: Macmillan.

Troscianko, J., Lown, A. E., Hughes, A. E., & Stevens, M. (2013). Defeating crypsis: detection and learning of camouflage strategies. *PLoS ONE, 8*, e73733.

von Helversen, B., Schooler, L. J., & Czienskowski, U. (2013). Are stripes beneficial? Dazzle camouflage influences perceived speed and hit rates. *PLoS ONE, 8*(4), e61173.

Zylinski, S., How, M. J., Osorio, D., Hanlon, R. T., & Marshall, J. (2011). To be seen or to hide: visual characteristics of body patterns for camouflage and communication in the Australian giant cuttlefish *Sepia apama*. *The American Naturalist, 177*(5), 681-690.

Zylinski, S., Osorio, D., & Shohet, A. J. (2009). Cuttlefish camouflage: context-dependent body pattern use during motion. *Proceedings of the Royal Society, Series B, 276*(1675), 3963-3969.
